# Sub-Optimal Compliance to Long-Term Inhalation Strategies and Poorer Health Care Outcomes Associated with Extended Tattoos in Adolescents with Mild-to-Moderate Bronchial Asthma

**DOI:** 10.3390/children11101254

**Published:** 2024-10-17

**Authors:** Roberto W. Dal Negro, Paola Turco, Massimiliano Povero

**Affiliations:** 1 National Centre for Respiratory Pharmacoeconomics and Pharmacoepidemiology—CESFAR, 37124 Verona, Italy; turcop@libero.it; 2 AdRes Health Economics and Outcomes Research, 10121 Turin, Italy; m.povero@adreshe.com

**Keywords:** bronchial asthma, adolescents, compliance to inhalation treatments, tattoos, impact on outcomes

## Abstract

Background: Patients’ compliance to inhalation therapy is usually sub-optimal in young asthmatics. Adolescents poorly compliant to regular anti-asthma treatments and those with tattoos (and the associated attitude) can share some personality traits and maladaptive behaviors. This relationship has not been investigated. Objective: To assess if “extended” tattoos can predict long-term compliance to regular therapy of adolescents with mild-to-moderate asthma. Methods: A 12-month retrospective observational investigation was conducted on non-smoker asthmatic adolescents of both genders. Patients assuming <70% of prescribed vilanterol/fluticasone furoate o.d. were defined as “non-compliant”. Tattoo surfaces were defined as “mild” or “extended” if they were < or ≥400 cm^2^, respectively. The relationship between tattoos and compliance on the evolution of resources consumption at 6 and 12 months was assessed by generalized estimating equation (GEE) models at the first and second semester of the treatment period. Results: It was found that 13.2% of compliant adolescents had mild tattoos, while 47.2% of non-compliant adolescents had mild-to-extended tattoos (odds ratio (OR) 6.91, 95% CI 2.49 to 19.17, *p* < 0.001). The mean annual adherence to treatment was 57.8% ± 10.1 SD expected doses in non-compliant subjects with “mild tattoos” (54.8 cm^2^ ± 36.9 SD), but 38.6% ± 11.4 SD expected doses in those with “extended tattoos” (568.4 cm^2^ ± 111.6 SD, *p* < 0.001). Total cost proved to be a linear trend from the lowest values of compliant patients with no/mild tattoos (EUR 65.22 at 6 months and EUR 33.63 at 12 months) to the highest values of non-compliant adolescents with extended tattoos (EUR 330.75 at 6 months and EUR 297.34 at 12 months). Conclusions: Tattoo extension might be used as a reliable predictor of poor compliance and higher health care costs in adolescents with mild-to-moderate asthma. Patients characterized by poor compliance to a long-term therapeutic strategy and tattooing attitude likely share some aspects of their personality profile.

## 1. Introduction

The inhalation route is of paramount importance in asthma management. However, there is consolidated evidence that the effectiveness of inhalation treatments is largely affected by patients’ compliance to the prescribed strategy, which is frequently sub-optimal in younger patients [1,2,3,4]. In general, therapeutic strategies requiring simple action plans and the lowest possible number of inhalations per day (i.e., one or two) usually encourage adherence to whatever anti-asthma strategy prescribed, particularly when fixed-dose LABA/ICS is delivered through a single device [5,6,7,8,9,10].

Unfortunately, adolescents with bronchial asthma are characterized by lower compliance to long-term treatments via inhalation when compared to adults, regardless of the molecules they use (such as both preventers and/or relievers) [11,12,13,14] and the frequency of prescribed daily inhalations [4,15].

Several factors commonly contribute to the lower effectiveness of adolescents’ asthma management, such as their (and their families’) insufficient knowledge of asthma, low awareness of asthma risks [16,17,18], and poor attitude to self-management [19,20].

Some personality traits and maladaptive behaviors of asthmatic adolescents can contribute to their poor adherence to regular anti-asthma therapy [21,22,23]. Curiously, similar psychological profiles and temperamental features also seem to underlie the attitude associated with tattooing among young people [24]. In particular, the presence of extended tattoos is regarded as a generic sign of psychological discomfort, particularly in adolescents who frequently tend to communicate their insecurity, dissatisfaction, and hostility through this attitude [25]. Despite several specific studies carried out in this particular field, no data are available at present, to the best of our knowledge, on the possible relationship between adolescents’ tattooing attitude and their compliance to long-term anti-asthma therapy via inhalation.

The objective of this study was to investigate if extended tattoos are related to the compliance of asthmatic adolescents to long-term respiratory treatments and corresponding health and economic outcomes.

## 2. Materials and Methods

A retrospective post-hoc observational investigation was carried out on 108 Caucasian adolescents with bronchial asthma managed between February 2018 and September 2019 at the Specialist Medical Centre (CEMS), Verona, Italy. These patients’ samples were recruited during a previous study that aimed to investigate the association between adherence to their prescribed medications, lung function, and corresponding health care outcomes; all criteria for these patients’ selection and inclusion are detailed elsewhere [15]. Briefly, major criteria for selection were as follows: non-smoker adolescents of both genders; Caucasian ethnicity; the presence of mild-to-moderate asthma; the absence of any relevant comorbidity (cognitive dysfunction included); and a prescription for (and presumable use of) fluticasone fumarate/vilanterol (FF/V) 90/22 mcg dry powder o.d. from a single device. Exclusion criteria were as follows: the presence of any condition that made inhalation difficult; unavailability of parents’ consent; insufficient clinical data; incomplete lung function; and the use of an ICS/LABA o.d. different from FF/V.

Gender, age, BMI, atopy, and lung function (such as forced expiratory volume in 1 s—FEV1; maximum mid-expiratory flow—MMEF; and maximum expiratory flow at 25% of lung filling—MEF25) were collected from all subjects at recruitment, and 6 months and 12 months after the survey, and corresponding values were reported as percent predicted. At these times, the MCh dose (in mcg) inducing a 20% drop from their FEV1 baseline value (PD20 FEV1) was also measured in all subjects. Moreover, n. exacerbations, school days off, GP and Specialist visits, and courses of systemic steroids and/or antibiotics were also calculated at recruitment, and after the first and second semesters of this study. Obviously, health care outcomes reported at recruitment (and to be compared to those measured at the 6th and the 12th month of the survey) were calculated over the six months before the index date of this study’s start.

As previously published [15], compliance to treatment was calculated as the percentage of inhalation doses/month skipped, such as the percentage of skipping days of treatment over each semester, because the inhaler contained thirty doses of drugs. As the device was provided with a dose counter, the adherence of each adolescent to the therapy was assessed monthly via telephone calls when all adolescents (or their parents) were requested to communicate to the interviewer the number of remaining doses visible in their device [15]. Based on the consolidated literature, patients who used <70% of prescribed doses were defined as “non-compliant” [26].

Patients were also classified according to the presence and extension of their tattoos. An overall extension of at least 400 cm^2^ (regardless of its variable distribution on the chest, neck, trunk, or arms) was defined as “extended” tattoos. All others were classified as “no” or “mild tattoos”, and their prevalence was calculated in each subgroup of subjects.

After each semester of this study, the total health care cost was calculated according to a societal perspective. It included both direct healthcare costs (i.e., due to visits, hospitalizations, and drugs) and indirect costs (parents’ loss of productivity due to adolescents’ school absenteeism). Drugs cost was calculated from the posology prescribed and their current reference prices, while the cost of visits and hospitalizations were calculated on the basis of the National tariffs. Finally, indirect costs were obtained as the n. days of school absenteeism due to asthma multiplied by the average value of the Italian daily salary, updated to 2023 [27].

### Statistics

Counts and percentages were used for categorical variables, while means and standard deviations (SD) were used for continuous variables. The non-parametric Wilcoxon–Mann–Whitney test was adopted to compare continuous baseline characteristics between compliant and non-compliant groups. The two-sided exact Fisher test was adopted for gender-related analysis.

The prevalence of tattoos in compliant and non-compliant adolescents was assessed by a logistic regression model adjusted for baseline characteristics, while the odds ratio (OR) and 95% confidence intervals (CIs) were used to assess the strength of association.

The impact of the interaction between tattoos and compliance on the evolution of resources consumption was estimated by means of a series of generalized estimating equation (GEE) models [28] (such as gamma family and identity as link function) with a covariate of treatment period (after the first and the second semester). The effects of the interaction between tattoos and compliance on health care outcomes were expressed as adjusted mean difference (AMD) and 95% CI.

All regression models included age and gender as covariates in order to adjust for patients’ characteristics. Statistic calculations were performed using STATA (StataCorp. 2017. Stata Statistical Software: Release 15. College Station, TX, USA: StataCorp LLC).

### Ethics

The original study (see Reference 16) was approved by the R&CG Ethical Committee during the session of 10 October 2017 (code: 02/RG02/2017). The present post-hoc analysis was approved by the same Ethical Committee during the session of 11 November 2021 (code: P01/RG05/2021).

## 3. Results

In the sample enrolled for the previous study, tattoos information was available for 106/108 adolescents. Baseline characteristics of compliant and non-compliant adolescents were well matched at enrolment (Table 1).

In the whole sample, the mean overall annual compliance to therapeutic strategies was 79.3% expected doses ±8.8 SD in compliant and 48.2% expected doses ±10.9 SD in non-compliant adolescents (*p* < 0.001), regardless of age and gender.

**Figure 1:**
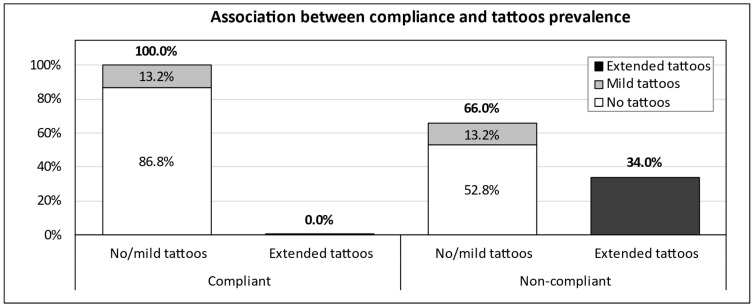
Distribution of tattoos among complaint and non-compliant adolescents.

Only 7/53 (13.2%) compliant adolescents had tattoos, while no extended tattoos were observed. Conversely, 7 subjects had mild tattoos (13.2%) and 18 (34.0%) had extended tattoos among the 53 non-compliant adolescents (Figure 1). The overall prevalence of tattoos was significantly higher in non-compliant than in compliant patients (47.2% vs. 13.2%, respectively, OR = 6.91, 95% CI 2.49 to 19.17).

In particular, when only non-compliant subjects were considered, their mean annual adherence to treatment was 57.8% ± 10.1 SD expected doses in subjects with “no/mild” tattoos, but 38.6% expected doses ±11.4 SD in adolescents with “extended” tattoos (*p* < 0.001). The mean tattoo surface was 54.8 cm^2^ ± 36.9 SD for patients with “mild tattoos”, while for those belonging to the group with “extended” tattoos it was 568.9 cm^2^ ± 111.6 SD (*p* < 0.001). No significant difference was found by age and gender.

**Figure 2:**
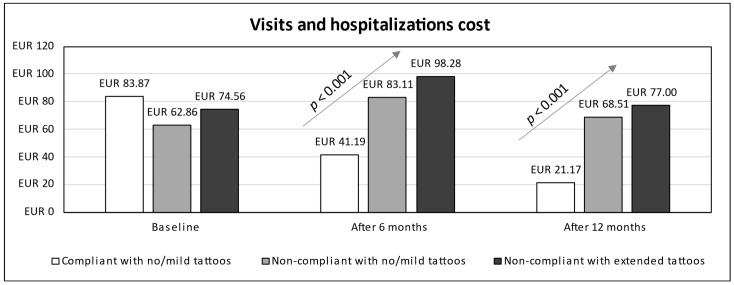
Time series for visits and hospitalizations cost (from baseline up to 12 months) stratified by compliance and tattoos prevalence.

**Figure 3:**
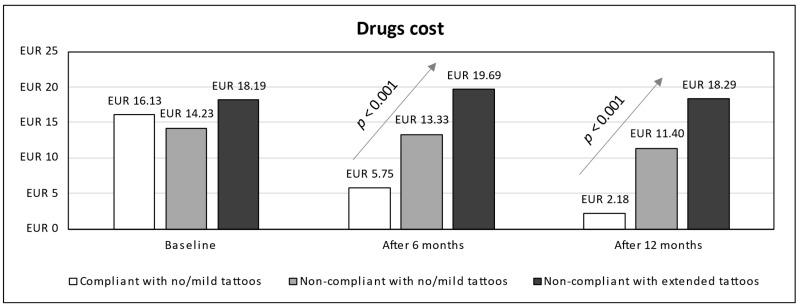
Time series for drugs cost (from baseline up to 12 months) stratified by compliance and tattoos prevalence.

**Figure 4:**
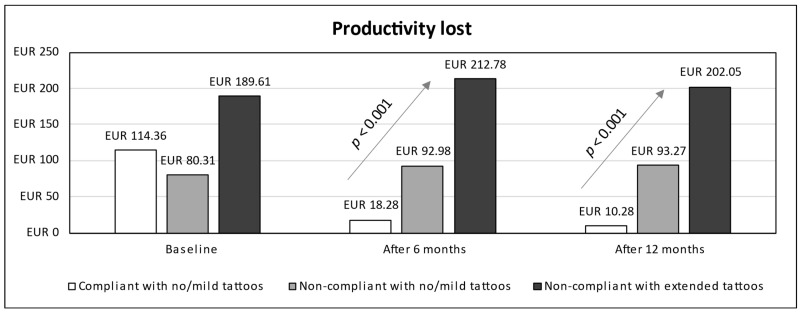
Time series for cost due to productivity loss (from baseline up to 12 months) stratified by compliance and tattoos prevalence.

**Figure 5:**
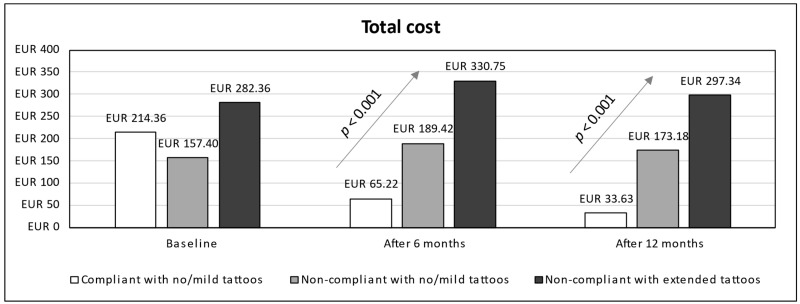
Time series for total cost (from baseline up to 12 months) stratified by compliance and tattoos prevalence.

In terms of cost analysis, subjects were grouped into three categories: compliant adolescents (all with no/mild tattoos), non-compliant adolescents with no/mild tattoos, and non-compliant adolescents with extended tattoos. Time series for estimated costs from baseline up to 12 months (stratified according to compliance and prevalence of tattoo categories) were detailed in terms of visits and hospitalizations costs (Figure 2), drugs cost (Figure 3), cost due to productivity loss (Figure 4), and total cost (Figure 5). A linear trend was shown in total cost from the lowest values for compliant patients with no/mild tattoos (EUR 65.22 at 6 months and EUR 33.63 at 12 months) to the highest values for non-compliant adolescents with extended tattoos (EUR 330.75 at 6 months and EUR 297.34 at 12 months).

Based on visual inspection of Figure 5 and results of GEE models (Table 2), the total cost for non-compliant adolescents with extended tattoos was higher than that calculated for compliant patients (AMD 66.2, 95% CI 7.5 to 124.8), while the cost for non-compliant adolescents with no/mild tattoos was lower (AMD −59.8, 95% CI −108.2 to −11.4). For compliant adolescents, a significant decrease in total cost was found over time, such as after both 6 (AMD −148.9, 95% CI −185 to −112.7) and 12 months (AMD −182.9, 95% CI −219.3 to −146.6). According to the interaction terms (time × patient category), non-compliance was associated with an increase in total cost at both 6 and 12 months, independently of the prevalence of tattoos. The same trend was observed for each cost item (visits and hospitalizations, drugs, and productivity lost).

## 4. Discussion

Since the 1970s, tattooing has become a popular practice that was, and still is, constantly growing among the general population, especially in youth. Its overall prevalence is supposed to be 10–20% in Europe, but 15–25% in young individuals, with variability according to the country and social/cultural status of subjects considered [29].

Further to the desire to merely follow the latest fashion trends (likely a generic sign of low charisma by itself), understanding the major motivations underlying the choice of the tattoo practice is crucial to the aim of identifying the reasons why individuals alter their body appearance. In general, the majority of studies tend to link the attitude of tattooing to peculiar personality traits of these subjects [25,30,31]. Although the search for individuality seems to act as one of the main driving forces [32], results of a large study carried out on more than four-hundred subjects confirmed that individuals who acquire tattoos mainly “tend to demonstrate self-consciousness and autonomy”, likely because these traits are self-perceived as deficient [25].

This attitude is particularly clear in young individuals, and adolescent in particular, who frequently complain of family conflicts, minor perceived support, and maladaptive behaviors [24]. On the other hand, an adolescent’s choice of tattoo has also been regarded as an expression of their mother–father relationship, and a symbolization of a taboo transgression [33]. Moreover, in these cases dissatisfaction (or/and hostility) with their family relationships has also been related to evidence that they frequently do not seek advice or inform their parents that they are getting a tattoo [34]. Actually, these aspects were suggested as valuable diagnostic indicators for their identity search and personality profile [25,35,36,37,38,39].

Indeed, similar psychological traits (such as anxiety, somatization, fear, insecurity, dissatisfaction, and hostility) have been described for some time as characterizing those asthmatic adolescents who have a frankly sub-optimal compliance to their regular anti-asthma strategy and management [11,12,13,14].

Curiously, these personality traits seem to largely correspond to those characterizing young patients with “extensive” tattoos.

Surprisingly, the relationship between “extended” tattoos and sub-optimal (or insufficient) compliance to long-term inhalation treatments has not been previously investigated, to the best of our knowledge, in adolescents with bronchial asthma, despite the somewhat common psychological profile underlying these two conditions.

In general, the basic overall degree of compliance results discriminate for the effective management of asthmatic adolescents, whatever the specific underlying cause (or causes). However, data from the present survey tend to confirm the strict relationship existing between the presence of “extended” tattoos and the degree of poor compliance to long-term anti-asthma strategies. Specifically, non-compliant patients with extended tattoos were associated with higher resources consumption, healthcare, and total costs than non-complaint patients with no/mild tattoos at baseline. However, the overtime cost increase was similar in the two groups at both 6 months (+EUR 181 vs. +EUR 196, *p* = 0.639) and at 12 months (+EUR 196 vs. +EUR 193, *p* = 0.947).

The roles of different causes of poor compliance to long-term anti-asthma treatment are unfortunately difficult to individually identify and assess in the majority of cases. As a consequence, the availability of a simple at-a-glance parameter (i.e., tattoos extension) would represent a helpful predictor of the future adherence of asthmatic adolescents to prescribed anti-asthma strategies.

The main weaknesses of the present study were as follows: (1) the survey was mono-centric and the sample was limited, though highly and carefully selected; (2) the threshold for tattoo extension was stated empirically; and (3) adolescents did not undergo individual psychological investigation, and their personality traits were assumed from the consolidated literature. This study’s main strengths were as follows: (1) the longitudinal survey was based on a certified large database; (2) all variables were extracted by Boolean equations according to inclusion/exclusion criteria and the planned times of this study; (3) adherence to treatment was carefully assessed monthly over the twelve-month survey; (4) sub-groups of compliant and non-compliant adolescents were well matched and comparable; (5) tattoo extension was carefully assessed in all subjects individually; and (6) this particular type of study has not been previously carried out.

## 5. Conclusions

Poor compliance to long-term therapeutic strategy and the tattooing attitude are presumed to be associated with some personality traits in asthmatic adolescents. Even though further specific studies are needed in order to confirm data from the present unprecedented study, it seems that the presence of “extended” tattoos might be used at a glance as a reliable predictor of poor compliance to long-term treatments and poorer health care outcomes in adolescents with bronchial asthma.

## Figures and Tables

**Table 1:** Baseline characteristics of enrolled patients.

Variables	Total	Compliant	Non-Compliant	*p*-Value
Patients	106	53	53	
Female (%)	42 (39.6%)	24 (45.3%)	18 (34.0%)	0.160
Median age (IQR)	16.0 (14.8–17.0)	15.5 (14.0–17.0)	16.0 (15.0–17.4)	0.050
Median FEV_1_ (IQR)	85.6 (75.4–95.0)	84.6 (75.0–95.8)	86.9 (78.1–96.8)	0.532
Median MMEF (IQR)	51.0 (39.2–64.7)	49.3 (32.1–55.8)	50.0 (39.2–63.3)	0.745
Median MEF_25_ (IQR)	43.0 (32.3–57.3)	43.1 (32.1–55.8)	41.9 (31.0–56.9)	0.953
Median PD_20_ FEV_1_ (IQR)	999.0 (411–1102)	998.0 (298–1070)	1020 (501–1192)	0.358

FEV_1_: forced expiratory volume in 1 s; IQR: interquartile range; MMEF: maximum mid-expiratory flow; MEF_25_: maximum expiratory flow at 25% of lung filling; PD_20_ FEV_1_: MCh dose (in mcg) inducing a 20% drop from the FEV_1_ baseline value.

**Table 2:** Effect of compliance, tattoos prevalence, and time dependency of total and detailed costs.

AMD (95% CI)	Visits and Hospitalizations Cost	Drugs Cost	Cost Due to Productivity Loss	Total Cost
**Variation in compliance ^1^**				
After 6 months vs. baseline	−42.7 (−65.8 to −19.6) ***	−10.4 (−14.1 to −6.6) ***	−99.6 (−126 to −73) ***	−148.9 (−185 to −113) ***
After 12 months vs. baseline	−64.9 (−82.3 to −47.5) ***	−14 (−17.7 to −10.4) ***	−110.3 (−140 to −80.4) ***	−182.9 (−219 to −147) ***
**Variation at baseline in non-compliance**				
In adolescents with no/mild tattoos vs. compliant adolescents ^1^	−22.7 (−43.4 to −1.9) *	−1.4 (−6.6 to 3.9)	−38.5 (−77.3 to 0.4) °	−59.8 (−109 to −11.4) *
In adolescents with extended tattoos vs. compliant adolescents ^1^	−11 (−32.4 to 10.3)	2 (−4.8 to 8.8)	72.1 (24.5 to 120) **	66.2 (7.5 to 125) *
**Interaction terms time** × **group** ^2^				
At 6 months in non-compliant adolescents with no/mild tattoos	62.7 (32.1 to 93.3) ***	9.4 (4.1 to 14.8) ***	108.8 (68.3 to 149) ***	181 (128 to 234) ***
At 6 months in non-compliant adolescents with extended tattoos	65.4 (29.9 to 101) ***	11.8 (3.5 to 20.2) **	119.3 (75.1 to 164) ***	196 (135 to 257) ***
At 12 months in non-compliant adolescents with no/mild tattoos	68.4 (46.6 to 90.1) ***	11.1 (5.2 to 17.1) ***	116.3 (70.8 to 162) ***	195.9 (143 to 249) ***
At 12 months in non-compliant adolescents with extended tattoos	65.2 (38.9 to 91.5) ***	14.1 (5.5 to 22.7) ***	115.8 (42.9 to 189) **	193.2 (113 to 273) ***
**Difference between interaction terms of non-compliant** adolescents **with extended tattoos vs. non-compliant** adolescents **with no/mild tattoos**				
	**Visits and hospitalizations cost**	**Drugs cost**	**Cost due to productivity loss**	**Total cost**
At 6 months	2.7 (−31.2 to 36.6)	2.4 (−6.0 to 10.8)	10.5 (−37.0 to 58.0)	15.0 (−47.8 to 77.9)
At 12 months	−3.2 (−27.5 to 21.1)	2.9 (−6.3 to 12.2)	−0.5 (−76.6 to 75.6)	−2.7 (−84.0 to 78.5)

^1^ with no/mild tattoos, ^2^ extra-variation from baseline in non-compliant adolescents with no/mild or extended tattoos. AMD: adjusted mean difference; CI: confidence interval. *** *p* < 0.001, ** *p* < 0.01, * *p* < 0.05, ° *p* < 0.1

## Data Availability

Data presented in this study are available on request from the corresponding author. Data are not publicly available due to privacy issues.
